# Evaluating the Effectiveness of Pharmacological Strategies and Further Measures for Pain Relief during Hysterosonosalpingography: A Systematic Review

**DOI:** 10.3390/diagnostics12123185

**Published:** 2022-12-16

**Authors:** Arianna Riva, Amerigo Vitagliano, Marco Noventa, Giovanni Buzzaccarini, Gianluca Raffaello Damiani, Antonella Vimercati, Danila Morano, Cristina Taliento, Pantaleo Greco, Ettore Cicinelli, Gennaro Scutiero

**Affiliations:** 1 Department of Women’s and Children’s Health, University of Padua, Via Nicolò Giustiniani 3, 35128 Padua, Italy; 2 Unit of Obstetrics and Gynecology, Department of Interdisciplinary Medicine (DIM), University of Bari, 70100 Bari, Italy; 3 Dipartimento di Scienze Mediche, Università Degli Studi di Ferrara, 44121 Ferrara, Italy

**Keywords:** hysterosonosalpingography, ART, IVF, pain relief, anaesthesia

## Abstract

This systematic review aims to evaluate the effectiveness of pharmacological and non-pharmacological strategies for pain relief in women during contrast-enhanced ultrasound for the assessment of tubal patency and uterine disease, compared with placebo or no intervention. In December 2021, we searched the electronic databases (Pubmed, Embase, Sciencedirect, the Cochrane library and Clinicaltrials.gov) without date restriction: We identified 10 randomized control trials (RCTs) (2098 women) eligible for this systematic review, after applying our inclusion and exclusion criteria. Among these, five studies compared the use of painkillers with the placebo, two studies compared different catheter positions in the cervix or in the uterine cavity, and two others considered different temperatures of the contrast medium, as a method to reduce pain. Topical lidocaine applied before the procedure may be associated with effective pain relief during hysterosonography, though the quality of this evidence is low. New echogenic contrast agents and their temperature at 37 °C ensure a less painful procedure. There is insufficient evidence to draw conclusions on the efficacy of other analgesics or strategies.

## 1. Introduction

Infertility is defined as inability to achieve a clinical pregnancy after 12 months or more of regular unprotected sexual intercourse [1]. It is a global public health issue affecting over 10% of women worldwide, with a pooled prevalence of 48 million couples and 186 million individuals [2,3,4]. In the female reproductive system, infertility may be caused by a range of abnormalities of the ovaries, uterus, fallopian tubes, and endocrine system. Tubal occlusion is responsible for 20% [5] of cases of female infertility, representing a major indication for IVF treatments. A strong suspicion of tubal inefficiency is raised in cases of previous genital infections (Chlamydia, Neisseria, etc.), pelvic endometriosis, pelvic adhesions, or uterine malformations. Nevertheless, tubal integrity cannot be assumed by the lack of a history of pelvic inflammation or reproductive system diseases. For these reasons, the thorough evaluation of tubal integrity (including the visualization of obstructions or inefficient tubal function) has become a standard part of the basic infertility workup and represents a guide to the clinical management of infertile couples.

Until recently, laparoscopic chromopertubation was the standard gold technique for the evaluation of tubal patency. During laparoscopic chromopertubation, methylene blue solution is instilled into the uterine cavity through a catheter. This solution should pass through the fallopian tubes into the abdominal cavity and thus become visible during laparoscopy. Although this technique is still offered during laparoscopic surgical procedures in women with infertility (e.g., endometriosis surgery, pelvic adhesiolysis after infections, myomectomy), its use for the exclusive evaluation of tubal patency is progressively reduced due to the patient’s risks and the need for general anesthesia [6]. During the last decades, various diagnostic methods have been tested to assess the tubal status and the uterine cavity at the same time. An ideal test should correctly identify the tubal or uterine disease with minimal false negative results. Further, the tests should be well-tolerated, cost-effective, easy to perform and free of complications. However, such an ideal investigation is yet to be found. Various X-ray- and ultrasound-based techniques with contrast media were developed, including hysterosalpingography (HSG), saline infusion sonography (SIS), and hystero-contrast sonosalpingography (HyCoSy) [7].

The advantages of ultrasound techniques as compared to HSG are the lack of radiation exposure and less patient discomfort. Moreover, these techniques are safe, feasible, and quick and can be offered in an outpatient setting. The diagnostic accuracy for detecting tubal occlusions is high for both HyCoSy and SIS (85% and 77%, respectively) [8]. 

The most common side effects of HyCoSy and SIS are pelvic discomfort, uterine cramping and vasovagal reactions. These symptoms are due to the cervical passage of the instruments, uterine distention, catheter misplacement, including contact with the fundal edge of the uterine cavity, and irritation of the peritoneal cavity with contrast media. The perceived pain is the result of the stimulation of pelvic splanchnic nerves (S2–S4) and hypogastric nerves (T10-L2) [9]. 

Several strategies have been proposed to reduce pain perception during a contrast-enhanced gynecological ultrasound, including the administration of different anti-inflammatory/antispasmodic drugs [10] and various modalities in the use of the instrumentation. Nevertheless, the evidence is controversial, and a summary of the evidence needs to be included. 

This systematic review of randomized controlled trials (RCTs) aimed to summarize the available evidence on the effectiveness of pharmacological strategies and measures to prevent procedure related pain during contrast-enhanced ultrasound for the assessment of tubal patency.

## 2. Materials and Methods

### 2.1. Search Strategy

A systematic literature search was conducted in electronic database (Pubmed, Embase, Sciencedirect, the Cochrane library and Clinicaltrials.gov) until December 2020 without date restriction. The search used specific key words and database indexing terminology. The key search terms included: tubal patency test OR contrast-enhanced gynecological ultrasound OR saline infusion sonography and hystero-contrast sonosalpingography [Mesh/Emtree] AND pain OR discomfort OR complications OR adverse events AND prevention OR relief OR control OR management OR reduction. 

### 2.2. Study Design

This is a systematic review of RCTs evaluating the effectiveness of pharmacological strategies and measures to prevent procedure related pain during contrast-enhanced ultrasound for the assessment of tubal patency. The review was reported following the Preferred Reporting Items for Systematic Reviews and Meta-Analyses (PRISMA) guidelines. 

### 2.3. Inclusion Criteria

- Population: Women undergoing HyCoSy or SIS for the assessment of tubal patency.
- Intervention: Pharmacological strategies and measures to prevent procedure related pain
- Comparator: Placebo or no intervention.
- Timing: Before or during HyCoSy or SIS.
- Outcome: Pain perception.

### 2.4. Study Selection and Data Extraction

Two authors (A.R., A.V.) independently screened titles and abstracts of studies obtained by the search strategy. The text of each potentially relevant study was obtained and assessed for inclusion in the review, independently by the two authors. A manual search of reference lists of retrieved studies and available review articles was successively performed to avoid missing relevant publications. The same authors (A.R., A.V.) also independently extracted data from studies about study features (design, setting, objectives, main findings), population characteristics (age, ethnicity, inclusion criteria), tubal patency tests (type of test, duration, volume of contrast media injected, type of intrauterine catheter), intervention (dose, timing and way of administration of pharmacological interventions, timing and modality of non-pharmacological interventions), and outcomes measurements (type of outcomes, methods for outcomes assessment, results). One other author (E.C.) independently reviewed the selection and data extraction process. The results were compared, and any disagreement discussed and resolved by consensus. According to the different interventions applied (i.e., pharmacological and/or non-pharmacological), each manuscript was systematically evaluated for inclusion in each section of our review.

### 2.5. Aim of the Systematic Review

To summarize available evidence on the effectiveness of (1) pain medication (placebo-controlled trials) and (2) descriptive research on factors influencing procedure-related pain during contrast-enhanced ultrasound for the assessment of tubal patency. 

### 2.6. Ethical Approval

As this study was a systematic review of published data, formal ethical approval was not required.

### 2.7. Data Synthesis and Analysis

We reported all descriptive characteristics of study including study design, year of publication, study setting, type and number of patients, type of tubal patency test, type of intervention, and study outcomes. Since there was a marked heterogeneity among studies in terms of the interventions and outcome measures reported, a quantitative data synthesis was not performed. 

### 2.8. Risk of Bias 

Two Authors (A.R., A.V.) independently assessed the methodological quality of included studies by using the criteria outlined in the Cochrane Handbook for Systematic Reviews of Interventions. Seven specific domains related to risk of bias were assessed: random sequence generation; allocation concealment; blinding of participants and personnel; blinding of outcome assessment; incomplete outcome data; selective data reporting; other bias. Authors’ judgements were expressed as “low risk”, “high risk”, or “unclear risk” of bias. For the estimation of “selective data reporting”, we evaluated study protocols, when available. If not available, studies were judged to present an unclear risk of bias. Results were compared and disagreements were resolved by consensus.

## 3. Results

### 3.1. Study Selection

The literature search based on our pre-defined key search item identified 3233 publications, after removing duplicates. The titles of these manuscripts were screened, resulting in 86 studies considered potentially eligible to be included in the review. Of the total of relevant manuscripts identified, 70 studies were excluded after the examination of the abstracts and 16 studies were further evaluated. After the evaluation of full text, four studies were additionally excluded: two manuscripts were review articles [11,12]; one study assessed exclusively the prevalence of UFs in pregnancy without evaluating their modifications [13]; one additional study [14] potentially reported the duplication of data included in another study. Finally, we identified 10 full text manuscripts eligible for this systematic review after applying our inclusion and exclusion criteria (Table 1).

### 3.2. Included Studies

- *Type of patients:*

Indication to the diagnostic procedure: six studies focused on infertile women [14,15,16,17,18,19,20]. The remaining four studies [21,22,23,24] included women referred to their institutions for different reasons. Specifically, in the study by Guney et al. [15], patients were suffering from infertility, menometrorrhagia or postmenopausal bleeding. Melcer et al. and Nirmal et al. [16,17] included infertile women. Spieldoch et al. [18] included women with infertility, recurrent miscarriage and abnormal uterine bleeding. In the work of Okzan et al. and Yung et al. [19,20], the indications to the diagnostic procedure were not specified. Regarding parity, patients were nulliparous in a single study [23]. In five studies, patients were mainly nulliparous (i.e., Young et al.: 80% and 95% in the intervention groups and 90% in the control group; Ahmadi et al.: 79.6% in the intervention group and 86% in the control group; Moro et al.: 72.1% in the intervention group and 74.5% in the control group; Spieldoch et al.: 66% and 71% respectively within intervention group and control group; Melcer et al.: 65% in the intervention group and 55.2% in the control group). In the study by Guney et al., patients were mainly pluriparous (i.e., 69.8% in the intervention group and 71.7% in the control group). In the studies by Fenzl et al., Okzan et al. and Nirmal et al., parity was not reported.

**Table 1:** General Features of the studies.

Author and Year	Study Design, Country and Time of Realization	Participants, Main Inclusion Criteria	Interventions	Catheter	Groups	Pain Perception	Outcomes
Moro F et al., 2012 [21]	RCT double-blind ------- Rome, Italy ------- January 2003-March 2010	856 infertile patients ----- -20–41 years ----- -Infertile for less than 3 years ----- Exclusion criteria: Hypersensitivity to hyoshine-N-butylbromide -AUB -acute sexual transmitted disease or pelvic inflammatory disease -severe male factor -known or suspected pregnancy -treatment with OCP	hysterosalpingo-contrast sonography (HyCoSy) ----- 30 min before 10 mg hyoscine- N-butylbromide tablet or placebo tablet	Foley catheter 6 Fr in diameter was inserted into the uterine cavity and the balloon of the catheter was inflated with 1.5–2 mL sterile saline ----- Air and saline solution were instilled (15 mL of each)	856 patients (n = 40 excluded) --- 408 Yoscine group and 408 placebo group	Stacey score (0–4) Operator asked after the procedure any pain experienced, comparing with pain suffered during menstrual cycle	-No difference in pain score -Differences in pain scoring according to fallopian tubal patency
Fenzl V., 2012 [22]	Prospective and randomized study ------- Croatia ------- Ignote period	138 patients ------- Infertile patients evaluated at the hospital ----- no exclusion criteria	First hypoechogenic (0.9% saline) following hyperechogenic (Echovist) contrasts at room (25 °C) or body temperature (37 °C) ----- All received 1 h before a tablet of 50 mg diclofenac	Foley catheter size eight was inserted in the istmic part of the uterine cavity; the balloon was filled with 1.5 mL sterile water	138 patients ------- n = 68 patients (25 °C) and n = 70 patients (37 °C)	VAS scores (0–10) immediately after the procedure	-Significant difference in pain during introduction of the same contrast at different temperature(*p* < 0.001) --- Echovist induces significantly less pain than saline infusion
Jareethum R et al., 2010 [23]	Double blind randomized controlled trial ------ Bangkok, Thailand ------- March 2009-December 2009	141 patients ------- -nulliparous women, -age over 18 years -never had HSG or hysteroscopy ----- Exclusion criteria: -sexually transmitted disease or pelvic inflammatory disease -abnormal pap smear -hypersensitivity to mefenamic or hyoscine	Saline infusion sonohysterography --- 30 min before procedure 2 tablets (=500 mg) of mefenamic acid, or 1 tablet of 10 mg of hyoscine or 1 or 2 tablets of a placebo	A non-balloon catheter with 6.6 Fr outer diameter ----- Normal saline (200 mL)	141 infertile patients(excluded 0) In 6 cases intervention failed --- Mefenamic group n = 46, Hyoscine group n = 47 and placebo group n = 48	VAS (0–10) -before starting -after catheter insertion -maximum pain during SIS -immediately after -30 min after	No statistically significant differences were found in baseline characteristics, pain and satisfaction scores
Guney M. et al., 2007 [15]	Double blind randomized controlled trial ------- Turkey --- September 2004- April 2006	120 patients ------- Unspecified inclusion criteria ------ Exclusion criteria: -pregnancy, -acute cervicitis, -or profuse vaginal bleeding.	Saline solution infusion sonohysterography (SIS) ----- 2%lidocaine 5 mL or saline solution 5 mL into the uterine cavity before SIS	A sterile balloon catheter was placed in the endocervix and inflated with 1 mL of sterile water (5–10 mL)	120 enrolled patients (n = 14 excluded) ----- 106 patients: Study group (n = 53), Placebo group (n = 53)	VAS pain scores (0–10)-and patients distress recorded by the physician during, immediately after and 20 min after	-Intrauterine lidocaine reduced pain in parous
Spieldoch R.L. et al., 2008 [18]	Prospective randomized clinical study ------- Wisconsin --- December 2004-August 2005	69 women ------- Exclusion criteria -previous hysterectomy -current pregnancy -active PID -patulous or stenotic cervical os	Saline solution infusion sonohysterography (SIS) ----- All patients took NSAIDs 1 h before procedure ----- Cervical or intrauterine balloon catheter	2 lumen 5 French balloon catheter distended with 1 mL	69 enrolled patients ----- n = 35 cervical catheter, n = 34 uterine catheter	VAS pain scores (0–10)at the time balloon was inflated and after deflation	-Nulliparous women had significantly more pain than did parous -they have less pain in the cervical placement upon inflation and similar upon deflation
Melcer Y. et al., 2021 [16]	Randomized double-blind placebo-controlled trial ----- Israel ----- June 2020- September 2020	85 infertile women ----- Exclusion criteria -allergic to lidocaine, -unprotected intercourse, -chronic pelvic pain, -profuse vaginal bleeding, -reported inflammation or infections of the genital tract -psychological or neurological lesions affecting sensation, -a history of cervical surgery, or cervical stenosis	Saline and foam gel consecutively sonohysterography 2D-3D ----- intrauterine infusion of 10 mL 2% lidocaine or 10 mL 0.9% normal saline solution before the procedure	balloon-less GIS catheter (GynaecologIQ, Delft, the Netherlands) with a soft tapered tip was inserted into the endocervical canal	85 women (n = 5 excluded) ----- n = 40 lidocaine group and n = 40 placebo group	VAS (0–10) before the procedure and pain during the phase of intrauterine foam instillation on the VAS scale while seated in the waiting area	VAS during the procedure indicated that lidocaine flushing was associated with significantly less pain than ratings in the saline group. In the nulliparous and multiparous lidocaine group of patients, significant pain relief was obtained by the use of lidocaine
Amhadi F et al., 2019 [24]	Randomized double blind 2-armed clinical trial ----- Iran ----- May 2012- May 2014	300 infertile women ----- 18–45 yo ----- Exclusion criteria: -acute pelvic infection -severe endometriosis -AUB -history of patulous or stenotic cervical os -large benign tumors or the cavity	Saline infusion sonohysterography ----- Cervical or intrauterine catheter balloon placement ----- No medications	6F balloon catheter into the cervical canal (mid cervix) or inside the uterine cavity; inflated with 1.2 mL of a sterile saline solution (10–30 mL).	348 women (n = 48 excluded) ----- n = 150 intrauterine balloon and n = 150 intracervical catheter	10 points visual analog scale at 2 periods: at time of inflation and after deflation	No statistical differences between the groups
Yung SSF et al., 2016 [20]	Randomized, double blind, placebo controlled trial ----- Hong Kong ----- February–June 2015	120 women ----- Aged 18 or over ----- Exclusion criteria: -history of cervical stenosis -Allergy to lidocaine -Pregnancy -acute cervicitis -profuse vaginal Bleeding	Saline contrast sonohysterography ----- Prior the procedure: -3 mL 2% lidocaine gel applied to the cervix and intrauterine infusion of 5 mL normal saline (lidocaine gel group) -3 mL gel lubricant applied to the cervix and intrauterine infusion of 5 mL 2% lidocaine (lidocaine infusion group) -3 mL gel lubricant	8-Fr infant feeding tube inserted into the uterine cavity	150 patients (n = 30 declined to participate) ----- n = 40 lidocaine gel group n = 40 lidocaine infusion group n = 40 placebo group	Pain score from 0 to 100 before, during and 20 min after the procedure	Topical lidocaine gel application and intrauterine lidocaine infusion do not further reduce pain levels
Ozkan S et al., 2016 [19]	Randomized controlled trial ----- Turkey ------ March 2011–August 2011	120 women ------ 23–62 yo ----- Exclusion criteria: -severe systemic medical conditions, -cervical stenosis, -acute cervicitis and/or vaginitis, -lidocaine allergy	Saline infusion sonography ----- Patients received -2 mL 2% lidocaine into the cervix at 4- and 8 o’clock positions at a depth of 2–3 cm paracervical block (group 2), or -an 18-gauge intravenous catheter into the cervical canal up to the internal os. Two milliliters of 2% lidocaine was injected into the uterine cavity paracervical block +intrauterine lidocaine (group 3), or -only saline (controls, group 1) Tenaculum was applied 5 min after All patients were prescribed 500 mg azithromycin as prophylaxis	number 4 carmen cannula was inserted in the uterine cavity. The uterine cavity was filled with 50 mL of normal saline solution	120 patients(n = 24 excluded) ----- 96 patients: -n = 32 saline (controls, group 1), -n = 32 paracervical block (group 2), or -n = 32 paracervical block +intrauterine lidocaine (group 3)	VAS scores (0–10). to grade discomfort experienced	-statistically differences between the saline and paracervical block groups, and between the saline and paracervical block + intrauterine lidocaine group. -no statistically significant difference between paracervical block and paracervical block + intrauterine lidocaine groups
Nirmal D. et al., 2006 [17]	RCT study double blinded ----- Cardiff, UK ----- unknown period	149 women ----- primary or secondary infertility ----- Exclusion criteria: -if laparoscopy was more appropriate -previous pelvic inflammatory disease -history suggestive of endometriosis -adnexal pathology	hysterosalpingo-contrast sonography (HyCoSy) ----- (Echovist) contrasts at room (25 °C) or body temperature (37 °C)	5 French gauge catheter via cervical canal was inserted into the uterine cavity with a small balloon at the tip of the catheter	149 patients ------- n = 77 patients (25 °C) and n = 72 patients (37 °C)	Stacey score (0–4) Operator asked after the procedure any pain experienced, comparing with pain suffered during menstrual cycle	-Significant reduction of pain perception when Echovist was warmed at 37 °C (*p* = 0.006)

Saline solution infusion sonohysterography (SIS); Abnormal uterine bleeding (AUB); hysterocontrastsonography (HyCoSy); Pelvic inflammatory disease (PID); Oral contraceptive pill (OCP); non steroidal anti-inflammatory drugs (NSAIDs); visual analogue scale (VAS).

*Diagnostic procedure*

1. Type of procedure: Three studies evaluated the tubal patency [17,21,22], while the others were conducted to control the uterine cavity. Melcer et al. studied tubal patency and endometrial cavity too. A preliminary pelvic examination was performed digitally [19,22], with B-mode transvaginal ultrasound [16,18,20,21] or with 3D transvaginal ultrasound [24], while two studies did not report any baseline examination [15,23]. Amhadi et al. and Yung et al. used a 3D ultrasound for evaluating intrauterine abnormalities, Ozkan et al. used a 4D examination.
2. Type of catheter: A wide heterogeneity in the catheter type and position characterizes these studies. Six studies utilized a balloon catheter, placed in the uterine cavity [21,22], in the cervix [15,16], or both [18,24]. Moreover, the catheter used measured 6 Fr in two studies [21,24], 5 Fr in Spieldoch et al. study, 8 Fr in Fenzl et al. study. Guney et al. and Melcer et al. did not state the catheter diameter. The balloon was filled with 1 mL of saline [15,18], 1.5–2 mL [21], 1.5 mL [22], 1.2 mL [24], and no information was given in the Melcer et al. study [16]. Four studies were conducted using a non-catheter balloon placed on the uterine cavity with different diameter: 6.6 Fr [23], 6 Fr [20], 5 Fr [17], 4 carmen [19].
3. Type of contrast medium: Six studies were performed using the saline infusion with different total volume: In the work of Jareethum et al., 200 mL was infused to each patient, Guney et al. used 5–10 mL, Amhadi et al. introduced 10–30 mL of saline solution; in the other three studies, the information was missing [16,18,20]. Moro et al. instilled air and saline solution (15 mL for each patient); Fenzl et al. used saline solution following positive contrast Echovist at maximum 10 mL of saline and 15 mL of Echovist. They also considered the temperature of the contrast media as at room (25 °C) or body temperature (37 °C). Melcer used normal saline or lidocaine followed by foam gel. Nirmal et al. used Echovist.
4. Other technical details: The procedures were performed during the follicular phase: late follicular phase [21], early follicular phase [24], before the 14th day [16] between 6th and 11th day [22], 6th and 10th [23], and on 8th and 9th days [20]. We cannot find any specification on timing exams in the studies of Guney et al., Nirmal et al. and Okzan et al. Fenzl et al. and Spieldoch et al. declared the patients had taken non-steroidal anti-inflammatory drugs1 h before the procedure. The mean time period of the complete procedure was considered only in the Moro et al. study. The tenaculum grasped the cervix in all patients of the Ozkan et al. study.
5. Type of intervention: Five studies compared the use of painkillers with the placebo [15,19,20,21,23], two studies compared different catheter positions in the cervix or in the uterine cavity [18,24]. Fenzl et al. and Nirmal et al. considered different temperatures of the contrast medium as a method to reduce pain. Moro et al. administered 10 mg of hyoscine-N-butylbromide tablet 30 min before the procedures. Jareethum et al. utilized 30 min before the exam 10 mg of hyoscine-N-butylbromide tablet or 500 mg of mefenamic acid. Four studies used lidocaine: Guney et al. provided lidocaine 2% in 5 mL into the uterine cavity prior the exam, Melcer et al. used 10 mL lidocaine 2% in the cavity before the foam injection, Yung et al. utilized lidocaine gel 2% applied to the cervix or lidocaine 2% in 5 mL into the uterine cavity, and Okzan et al. used paracervical block with or without lidocaine 2% in 2 mL into the uterine cavity.

*Outcomes measures*

The primary aim was the evaluation of the pain perception using a 10 points visual analog scale (VAS), except for two studies, where the pain scoring was measured by Stacey score from 0 to 4 [21] or from 0 to 100 [20]. The authors evaluated any pain experienced at various times: immediately after the procedure [21,22], before the exam’s start and during the foam installation [16], during the exam, immediately after and later (20 min after, Guney et al.; 30 min after Ozkan et al.), or during the procedure and 20 min after [20], before starting, after the catheter insertion, during the exam, immediately and 30 min after the procedure [23], or at the time the balloon catheter was inflated and after deflation [18,24]. Nirmal et al. did not specify the exact moment when the whole procedure was evaluated.

*Pharmacological strategies for prevention of procedure related pain*

The interventions on pain relief were led by antispasmodic drugs or painkillers. Two studies used an antispasmodic drug: 10 mg of hyoshine-N-butylbromide was administered before the procedure and compared with placebo [21] or mefenamic acid and placebo in Jareethum’ study [23]. In the first study, there was no statistically significant difference in pain score between the two groups during the procedure, even if significant differences were found in pain scoring according to Fallopian tubal patency, regardless of treatment group (*p* < 0.0001). In the second study, the pain scores at every point were not statistically significantly different among groups: before the exam *p* = 0.810, after catheter insertion *p* = 0.540, during the exam *p* = 0.755, immediately after *p* = 0.771, 30 min after the exam *p* = 0.503. In this study the tubal patency was not evaluated, and the total volume of saline solution was higher (200 mL) than in the Moro study (15 mL), where the exam was also performed with air.

Four studies investigated the effect of different administration of lidocaine before the exam [9,10,13,14]. Guney et al. [15] performed sonohysterography with endocervical balloon and gave 2% lidocaine in 5 mL of saline solution or placebo: they determined significant lower pain, when lidocaine was used, in main pain scores during, immediately after and 20 min after the procedure (22%, 21.5%, 26.8%, *p* < 0.001). In the nulliparous patients, analyzed separately, no significant pain relief was obtained by the lidocaine (4.88 +/− 1.01; 3.88 +/− 0.86; 3.22 +/− 0.55; *p* > 0.05). Melcer et al. [16] performed the same protocol during hysterosalpingo-foam sonography exams with a balloon-less GIS catheter with a soft tapered tip inserted into the endocervical canal: in the same way, they found reduced pain perception during the procedure in the lidocaine group (3.0 +/− 1.3 vs. 6.3 +/− 1.5, *p* = 0.001). The incidence of severe pain was significantly lower in the lidocaine group than the saline group (2.5% and 45%, *p* = 0.001). In the nulliparous lidocaine group of patients, significant pain relief was lower with the use of lidocaine (3.0 +/− 1.4 vs. 6.3 +/− 1.5, *p* = 0.001). Ratings on the VAS in patients who were multiparous found lower pain (3.0 +/− 1.3 vs. 6.2 +/− 1.4, *p* = 0.001). Young et al. [20] during sonohysterography with intrauterine balloon looked at the effect of 3 ml 2% lidocaine gel on the cervix and 5 mL of saline in the uterine cavity or 3 mL gel lubricant on the cervix and intrauterine infusion of 5 mL 2% lidocaine intrauterine or placebo in the cervix and in the uterine cavity. They failed to find statistical differences in any case (after speculum insertion *p* = 0.92, after test solution infusion *p* = 0.72, during normal saline infusion *p* = 0.80, immediately after *p* = 0.96, 20 min after *p* = 0.33, overall pain score *p* = 0.14). When nulliparous and parous patients were analyzed separately, they did not find significant differences in pain scores. Okzan et al. [19] during saline infusion sonography confronted different types of paracervical block before grasping the cervix with a tenaculum: patients were randomized in three groups, where 2 mL 2% of lidocaine was injected on the cervix (PCB group) or 2%lidocaine was added into the uterine cavity (PCB + IUL group) or saline solution only was used. They found significant differences among groups at tenaculum placement: pain scores were significantly higher in the control group (*p* = 0.002, 95% CI 4.33–21.91), but there were no significant differences between PCB and PCB + IUL groups (*p* = 0.596, 95% CI −5.20–12.38). After the instillation of saline solution, the control group had significantly more pain perception than PCB group (*p* = 0.045, 95%CI 0.28–29.15) and PCB + IUL group (*p* = 0.01, 95%CI 3.75–32.6), but there were no differences between PCB and PCB + IUL groups (*p* = 0.835, 95% CI −10.9–17.9).

*Measures for prevention of procedure related pain*

Fenzl et al. [22] evaluated the effect of different temperatures in two distinct contrasts media, and they showed a significantly less pain with Echovist (3.91 vs. 2.37, *p* < 0.001) in comparison to sterile saline (4.99 vs. 3.43, *p* < 0.001). Between these two groups, they also found statistically significant differences in pain during introduction of the same contrast at different temperatures: contrast at 37 °C was more tolerable (3.43 vs. 2.37, *p* = 0.001) than the contrast at 25 °C (4.99 vs. 3.91, *p* = 0.002). Nirmal et al. [11] assessed the two different temperatures of Echovist (25 versus 37 °C) in infertile patients, proving a significant reduction in pain perception when contrast was at 37 °C (3.86 vs. 5.1, *p* = 0.006).

Spieldorch et al. and Amhadi et al. [18,24] assessed during sonohysterography the pain perception based on the catheter position: in Spieldorch et al. study, VAS score was lower (1.8 +/− 2.1 vs. 3.0 +/− 2.3, *p* = 0.02) in patients with intracervical catheter during the inflation of the balloon, while after balloon deflation, perceived pain did not differ (2.2 +/− 2.4, 2.0 +/− 2.4, *p* = 0.66). The total volume of saline solution used to complete the exam was significantly lower with cervical placement (19 +/− 16 vs. 40 +/− 32 mL, *p* = 0.001). Moreover, nulliparous women had significantly more pain than parous women after balloon inflation (2.7 +/− 2.4 vs. 1.6 +/− 1.9, *p* = 0.04) and after deflation (2.5 +/− 2.5 vs. 1.3 +/− 1.7, *p* < 0.05). Nulliparous women reported less pain with cervical balloon than uterine balloon (1.4 +/− 1.7 vs. 3.6 +/− 2.5, *p* = 0.001). In the Amhadi et al. study, the pain perception did not statistically differ between the two groups. The total volume of saline solution used was lower in the cervical group when compared with cavity catheter placement in the treated group (24.2 +/− 0.9 vs. 27.4 +/− 0.9 mL, *p* = 0.015). The pain perception in nulliparous patients compared to parous women did not differ after inflation of the balloon (1.6 +/− 0.1 vs. 1.1+/− 0.2, *p* = 0.069) or after deflation (0.4 +/− 0.1 vs. 0.5+/− 0.1, *p* = 0.874).

Materials and methods are available as supplementary materials.

## 4. Discussion

In order to decrease unpleasant experiences for our patients, we first carried out a literature review on pharmacological strategies and measures for pain relief during hysterosonosalpingography. Indeed, we realized that even published guidelines on hysterosonosalpingography lack concrete indications on how to prevent or restore pain [25,26]. More accessible instructions would be helpful for daily practice, because this exam has several benefits, such as its use in an office setting without radiation exposure. Furthermore, technical advances have been made to reduce patient discomfort by developing thinner catheters and new, higher performing contrast media. Besides these signs of progress, women consider, by tradition, hysterosonosalpingography a painful procedure, being emotionally proven even before starting the exam.

The sparsity and heterogeneity of evidence from the literature determine huge variations in clinical practice and vice versa. The topic is challenging because pain is influenced by culture and ethnicity, and is composed of sensory, emotional, and cognitive components. It is triggered by uterine distention, consequent contractions, stimulation of the nerves of the inferior hypogastric plexus, stimulation of nerve fibers or the cervix (with a balloon catheter, or tenaculum grasp), or peritoneal irritation. These topics are extensively discussed in the case of office hysteroscopy [27,28,29], but in the case of hysterosonosalpingography several recent RCTs have failed to demonstrate a significant benefit of pharmacological and factor influencing procedure related pain.

Three studies assessed intrauterine transcervical local anesthesia. Whereas one randomized study [16] proved that 10 mL of 2% lidocaine flushing in the uterine cavity is effective for hysterosonosalpingography-related pain, lidocaine 5 mL distension medium [15] was effective in another only in parous women. Yung et al. showed that neither topical lidocaine gel nor an intrauterine 5 mL of 2% lidocaine infusion reduced pain during the procedure.

One limitation of the studies that assess the instillation of lidocaine in the uterine cavity is that they need to take into account the duration of the procedure and, consequently, the nerve stimulation time. Moreover, speculum placement is a painful part of the procedure and could limit the beneficial effect of lidocaine infusion. Another issue is the use of an infant feeding tube instead of the balloon, which is responsible for the inferior pain perceived generally and in the control group too in the Young study: the lower pain recorded could be the reason why no significant difference was found and may suggest an important role of the cervical innervation from parasympathetic fibers from the pelvic splanchnic nerves.

Two studies [21,23] failed to find a benefit from administering 10 mg orally hyoscine-N-butylbromide, an anticholinergic drug, 30 min before the procedure. Furthermore, a study [30] conducted during conventional uterine magnetic resonance MR images showed that the anticholinergic agents significantly suppressed sporadic myometrial contractions and uterine peristalsis. This discrepancy could be easily explained by the inability to counteract the distension induced by the saline solution. Moro et al. noticed a relationship between pain perception and tubal obstruction, probably due to an over distension of the uterine muscular fibers and higher uterine contractility, which cannot benefit from antispasmodic drugs.

With regard to the catheter’s characteristics, we found two studies comparing its position in the cervix or the uterus. Whereas one prospective randomized trial [18] established lower pain perception during the initial part of the procedure with the intracervical balloon placement, another [24] failed to demonstrate differences in pain perception between the two groups. The pain perceived during the exam may be due to the mechanical distension of the uterine wall and the stimulation of mechanoreceptors located on the muscle layer. No studies have confronted different catheter types (balloon vs. non-balloon) or diameters.

The contrasting temperature was investigated by Fenzel and Nirmal et al. RCTs. The temperature of the media is shown to be a causative agent of pelvic pain and contrasts of body temperature (37 °C) are better tolerated than those of room temperature (25 °C). Heat presents an anti-irritation action to inhibit nociceptive stimuli and decrease sympathetic nervous activity. It presents an antispasmodic and vasodilatory role. A preheated saline solution to distend the intrauterine cavity could be a valid option to reduce pain, while avoiding stimulating uterine wall contractility [31].

## 5. Conclusions

Hysterosonosalpingography is a simple, cost-effective, and easy to perform procedure in the outpatient setting. Topical lidocaine, as applied before the procedure, may be associated with effective pain relief during hysterosonography, though the quality of evidence is low. New echogenic contrast agents and their maintenance at 37 °C may ensure a less painful procedure. There is insufficient evidence to draw conclusions on the efficacy of other analgesics or strategies.

## Data Availability

All data are provided within this document.

## Supplementary Materials

The following supporting information can be downloaded at: https://www.mdpi.com/article/10.3390/diagnostics12123185/s1, Table S1: suggested greed for hysterosonosalpingography.
