# Supplementary material for: Evaluating the Effectiveness of Pharmacological Strategies and Further Measures for Pain Relief during Hysterosonosalpingography: A Systematic Review
Source: Diagnostics (Basel). 2022 Dec 16;12(12):3185. doi: 10.3390/diagnostics12123185 (PMC9777723; doi:10.3390/diagnostics12123185)
Supplement: Supplementary file 1 [file diagnostics-12-03185-s001.zip › diagnostics-1987241-supplementary.pdf]

Supplementary table 1

Practice recommendations for performing hysterosonosalpingography:

1) Choose a comfortable setting, spending some time on giving the right information on the technical approach

2) Bladder filling may be a simple recommendation in case of anteverted uterus to reduce the angle between uterine cervix and body and facilitate the catheter placement

3) Instillation of 5 ml 2% lidocaine into the uterine cavity before the procedure is a cheap and easy painless strategy

4) A thin catheter without a balloon may reduce the early contractions of the uterus and the cervical nerves stimulation. Tenaculum grasp use may be avoided.

5) New echogenic contrast agents made of suspension of polymers seem to streamline the technique, causing less pain than saline infusion.

6)Available evidence does not allow drawing conclusions about a limit of contrast medium quantity that should not be exceeded, but in general the lower the volume, the lower the risk of discomfort and pain.

7) Warm contrast agent at 37°C is associated with a less painful procedure.

Supplementary table I: suggested greed for hysterosonosalpingography

**Expert opinion recommendation**	**Evidence based recommendation**
Choose a comfortable setting, spending some time on giving the right information on the technical approach	Instillation of 5 ml 2% lidocaine into the uterine cavity before the procedure is a cheap and easy painless strategy
Bladder filling may be a simple recommendation in case of anteverted uterus to reduce the angle between uterine cervix and body and facilitate the catheter placement	A thin catheter without a balloon may reduce the early contractions of the uterus and the cervical nerves stimulation. Tenaculum grasp use may be avoided
	New echogenic contrast agents made of suspension of polymers seem to streamline the technique, causing less pain than saline infusion
	Available evidence does not allow drawing conclusions about a limit of contrast medium quantity that should not be exceeded, but in general the lower the volume, the lower the risk of discomfort and pain.
	Warm contrast agent at 37°C is associated with a less painful procedure
